# TB-IRIS: Proteomic analysis of *in vitro* PBMC responses to *Mycobacterium tuberculosis* and response modulation by dexamethasone

**DOI:** 10.1016/j.yexmp.2017.02.008

**Published:** 2017-04

**Authors:** Liam Bell, Janique M. Peyper, Shaun Garnett, Rabecca Tadokera, Robert Wilkinson, Graeme Meintjes, Jonathan M. Blackburn

**Affiliations:** a Department of Integrative Biomedical Sciences & Institute of Infectious Disease and Molecular Medicine (IDM), University of Cape Town, South Africa; b Centre for Proteomic and Genomic Research (CPGR), Observatory, 7925 Cape Town, South Africa; c Department of Medicine, Imperial College, London W2 1PG, UK; d Clinical Infectious Diseases Research Initiative, IDM, University of Cape Town, Observatory, 7925 South Africa; e Department of Medicine, University of Cape Town, Observatory, 7925 South Africa; f Francis Crick Institute, Mill Hill Laboratory, London NW7 1AA, UK; g HIV/AIDS, STIs and TB Programme, Human Sciences Research Council, Arcadia, 0002 Pretoria, South Africa

**Keywords:** Tuberculosis-HIV co-infection, Anti-retroviral therapy, Paradoxical tuberculosis-associated immune reconstitution inflammatory syndrome, Human peripheral blood mononuclear cells, Proteomics, Mass spectrometry

## Abstract

Paradoxical tuberculosis-associated immune reconstitution inflammatory syndrome (TB-IRIS) occurs in 8–54% of South African patients undergoing treatment for tuberculosis/human immunodeficiency virus co-infection. Improved TB-IRIS molecular pathogenesis understanding would enhance risk stratification, diagnosis, prognostication, and treatment. We assessed how TB-IRIS status and dexamethasone influence leukocyte proteomic responses to *Mycobacterium tuberculosis* (Mtb).

Patient blood was obtained three weeks post-anti-retroviral therapy initiation. Isolated mononuclear cells were stimulated *ex vivo* with heat-killed Mtb in the presence/absence of dexamethasone. Mass spectrometry-based proteomic comparison of TB-IRIS and non-IRIS patient-derived cells facilitated generation of hypotheses regarding pathogenesis.

Few represented TB-IRIS-group immune-related pathways achieved significant activation, with relative under-utilisation of “inter-cellular interaction” and “Fcγ receptor-mediated phagocytosis” (but a tendency towards apoptosis-related) pathways. Dexamethasone facilitated significant activation of innate-related pathways. Differentially-expressed non-IRIS-group proteins suggest focused and co-ordinated immunological pathways, regardless of dexamethasone status.

Findings suggest a relative deficit in TB-IRIS-group responses to and clearance of Mtb antigens, ameliorated by dexamethasone.

## Highlights

• TB-IRIS immunopathogenesis was investigated *via* mass spectrometry-based proteomics.
• TB-IRIS status and dexamethasone altered leukocyte response to *Mycobacterium tuberculosis*.
• Altered leukocyte proteomic responses suggest mechanisms underlying TB-IRIS.
• Dexamethasone ameliorates potential deficits in antigen clearance.

## 1 Introduction

*Mycobacterium tuberculosis* (Mtb) is the most prevalent human immunodeficiency virus (HIV)-associated opportunistic infection (OI), with up to 70% of new TB cases in South Africa (SA) being HIV co-infected (Leone et al., 2010) and with Sub-Saharan Africa accounting for ~ 80% of the global burden of HIV-associated TB (Lawn and Churchyard, 2009). Although antiretroviral therapy (ART) reduces the risks of active TB and HIV-related mortality (Harries et al., 2009, Meintjes and Lynen, 2008), combined treatment is associated with various complications, including tuberculosis-associated immune reconstitution inflammatory syndrome (TB-IRIS) (Leone et al., 2010, Harries et al., 2009, Schutz et al., 2010).

Immune reconstitution inflammatory syndromes occur during recovery from relative immunosuppression in the presence of an OI (Lai et al., 2013, Meintjes et al., 2008a, Barber et al., 2012, Tan et al., 2011, Singh and Perfect, 2007, Lawn et al., 2005, Price et al., 2001), most commonly after ART initiation in the presence of Mtb infection. This study focuses exclusively on paradoxical TB-IRIS (henceforth ‘IRIS’), wherein patients previously improving on TB treatment experience worsening of TB symptoms a median of 14 days after ART initiation (Meintjes and Sonderup, 2011). The incidence of IRIS varies from < 10% to > 50% (Meintjes and Lynen, 2008, Narendran et al., 2013, French, 2007, Breton et al., 2004, Shelburne et al., 2005, Michailidis et al., 2005, Manosuthi et al., 2006, Lawn et al., 2007, Burman et al., 2007, Namale et al., 2015, Breen et al., 2004, Olalla et al., 2002) and a meta-analysis estimates the mortality rate at 3.2% (Burman et al., 2007, Namale et al., 2015), with an average symptom duration of two to three months (although symptoms can persist for years) (Meintjes et al., 2008a, Barber et al., 2012, Tan et al., 2011, Singh and Perfect, 2007, Lawn et al., 2005, Price et al., 2001, Meintjes and Sonderup, 2011, Narendran et al., 2013, French, 2007, Breton et al., 2004, Shelburne et al., 2005, Michailidis et al., 2005, Manosuthi et al., 2006, Lawn et al., 2007, Burman et al., 2007, Namale et al., 2015, Breen et al., 2004, Olalla et al., 2002). Known risk factors include disseminated and extrapulmonary TB (Breton et al., 2004, Manosuthi et al., 2006, Burman et al., 2007, Namale et al., 2015), short interval between TB treatment and ART initiation (Burman et al., 2007, Namale et al., 2015, Breen et al., 2004, Olalla et al., 2002), vigorous immunological and virological responses to ART (Breton et al., 2004) and low CD4 count prior to ART initiation (Michailidis et al., 2005, Manosuthi et al., 2006, Lawn et al., 2007). Underlying mechanisms are incompletely understood, but high antigen load and exaggerated inflammatory responses on immune reconstitution appear to be central (Shelburne et al., 2005, Grant et al., 2010, Murdoch et al., 2008, Manabe et al., 2007). Although delaying ART reduces the risk of IRIS, it unacceptably increases HIV-related mortality when CD4 count is < 50 (Uthman et al., 2015). Current SA guidelines (Meintjes and Maartens, 2012) thus recommend that urgency of ART in TB patients is dictated largely by immune (CD4) status.

IRIS is thought to arise *via* a complex interplay between residual Mtb antigens and rapidly reconstituting immunity (Kwan and Ernst, 2011). Pathogenesis and natural history are incompletely understood, but hypotheses implicate the same immune mediators - including macrophages, IFNγ, TNFα, IL-6, and CD4 + T-cells - required for TB control (Lai et al., 2013, Meintjes et al., 2008a, Barber et al., 2012). IRIS may co-occur with a Th1 population expansion (Bourgarit et al., 2009, Meintjes et al., 2008b), and is associated with hypercytokinemia (including IFN-γ) and restoration of delayed-type hypersensitivity to Mtb antigens (Tadokera et al., 2011, Schluger, 2002, French et al., 1992). ART restores the host granulomatous response to mycobacteria (Lawn et al., 2004, Bartley et al., 1999). IRIS predisposition is also associated with inappropriate control of complement activation (Tran et al., 2013).

Immune restoration, perhaps coupled with relatively lacking immunoregulation, may lead to IRIS-characteristic tissue-damage (Lawn et al., 2005). Based on murine models, Barber et al. (2012) proposed that following lymphopaenic uncoupling of innate and adaptive responses, the timing of macrophage infection with Mtb - relative to CD4 + T-cell-mediated activation of the infected macrophage - is a critical factor in IRIS development: hyper-activation of accumulated primed macrophages may lead to inflammatory tissue destruction.

There is currently no validated immunological marker of IRIS to guide clinical management: instead, identification is largely by clinical diagnosis of exclusion (Meintjes et al., 2008a). A double-blinded, randomized, placebo-controlled study (Meintjes et al., 2010) demonstrated that prednisone reduced paradoxical IRIS morbidity, concurrent with reductions in serum IFNγ, TNFα, IL-6, IL-12, and IL-10, suggesting an effect mediated *via* reduced cytokine effector responses (Maertzdorf et al., 2011, Skolimowska et al., 2012). At clinical doses, glucocorticoids are known to have immunosuppressive and anti-inflammatory effects (Zen et al., 2011). However, while prednisone ameliorates IRIS symptoms, it may not resolve underlying pathogenic mechanisms. Better understanding of the molecular basis of IRIS would therefore inform improved risk-stratification, diagnosis, prognostication, and definitive prevention and/or treatment strategies. Clinical and immunological data suggest a difference in *in vivo* inflammatory responses in persons predisposed to IRIS. Here, we have assessed whether IRIS status influences immune cell proteomic response to Mtb, as well as how dexamethasone modulates these responses, in order to provide insight into the molecular pathogenesis.

## 2 Materials and methods

### 2.1 Study design

This was a pre-clinical mass spectrometry-based differential proteomic profiling study to assess whether IRIS status influences immune cell proteomic response to Mtb, and how corticosteroids modulate this response. PBMC from IRIS (*n* = 30) and non-IRIS (*n* = 30) participants were cultured *ex vivo*, mimicking *in vivo* conditions of IRIS development and symptom alleviation (stimulation with Mtb ± dexamethasone, in the presence of first-line ART). Global protein expression differences were interrogated to generate hypotheses regarding the molecular mechanisms underlying IRIS and its symptom alleviation by corticosteroids. The sample size (*N* = 60) provides > 95% power to detect a two-fold change in expression (*p* < 0.01).

### 2.2 Ethics, patient recruitment, and sample acquisition

This study was nested within a parent study entitled “Prospective cohort study of hospitalised HIV-TB co-infected patients at Brooklyn Chest Hospital” (van der Plas et al., 2013), approved by the University of Cape Town Human Research Ethics Committee (HREC 049/2009). All participants provided written informed consent.

**Fig. 1:**
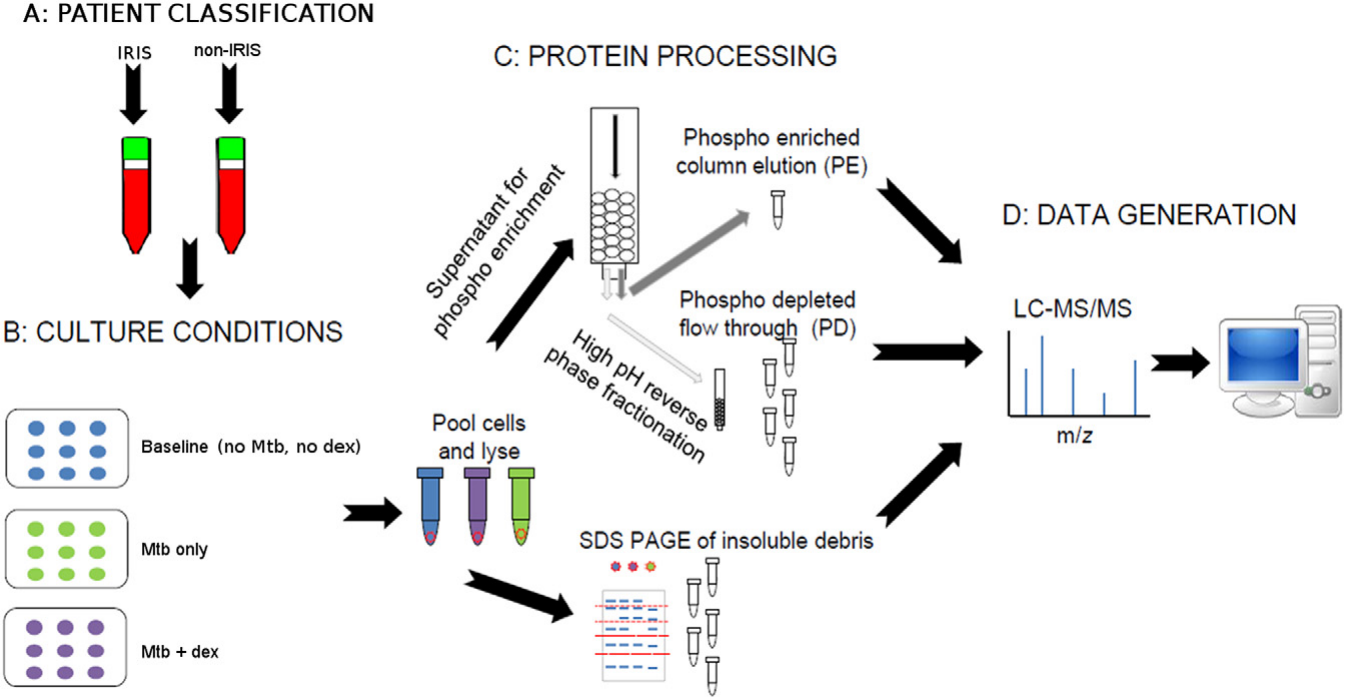
Experimental workflow. (A) PBMC derived from co-infected patients (post-ART initiation) were classified as IRIS or non-IRIS. (B) PBMC were cultured under four conditions (baseline and three test conditions) prior to harvesting, pooling, lysis, and centrifugation. Supernatant was phospho-enriched, and the phospho-depleted fraction separated into five fractions by high pH reverse-phase fractionation. (C) The pellet was re-suspended and separated into five fractions *via* SDS-PAGE. (D) Eleven resultant fractions per group were analysed by label-free LC-MS/MS. (E) Crux-Barista employs the SEQUEST PSM algorithm to assign peptide identities to mass spectra, in order to identify and relatively quantitate (by NSAF) proteins.

As previously described, hospitalised ART naïve HIV-TB patients meeting eligibility criteria were recruited and followed for development of paradoxical TB-IRIS using a consensus clinical case definition (Meintjes et al., 2008a). Venous blood samples were obtained at three weeks post-ART initiation, and subsequently classified into two groups: ‘IRIS’ or ‘non-IRIS’ (Fig. 1a). Samples were transported at room temperature to a BSL2 facility at the Institute of Infectious Diseases and Molecular Medicine within 4 h. Exhaustive cell counts were performed at every step of downstream sample processing.

### 2.3 PBMC isolation

PBMC from 30 IRIS and 30 non-IRIS samples were isolated using standard Ficoll-histopaque (GE Healthcare Bio-Sciences AB) density gradient centrifugation. PBMC were stored at − 80 °C in cryoprotective medium (RPMI-1640 [Sigma-Aldrich], 10% fetal calf serum [FCS; Cell Technology Inc], 10% dimethylsulfoxide [DMSO; Sigma-Aldrich]). RPMI containing 10% FCS is hereafter referred to as R-10.

### 2.4 PBMC culture

To avoid batch effects, samples were randomized to processing blocks. PBMC were rapidly thawed to 37 °C in blocks of ≤ four vials, and DMSO-containing media was immediately exchanged for R-10 by centrifugation (600 *g*, 10 min, room temperature). Cells were rested overnight (37 °C, 5% CO_2_), washed twice (as above), re-suspended in R-10, and plated (100,000 PBMC/200 μL/well) in sterile 96-well round-bottomed suspension plates (Costar, Corning).

PBMC culture conditions (Fig. 1b) mimicked *in vivo* conditions under which IRIS develops. To each well, the following were added: first-line ART cocktail to final physiologically relevant concentrations of 9 μM lamivudine (3TC; NIH, Germantown, USA), 115 nM efavirenz (EFV, NIH), and 5 μM zidovudine (AZT, NIH); heat-killed Mtb H37Rv to a final multiplicity of infection (MOI) of 5:1; and, where appropriate, dexamethasone to a final physiologically relevant concentration of 15 ng/mL. PBMC from each participant were cultured (37 °C, 5% CO_2_) independently for 20 h (the time of maximal differential gene expression, as determined by qPCR; data not shown) under the following three conditions: no stimulus (baseline), plus Mtb only, and plus Mtb *and* dexamethasone.

Cells from each participant were harvested, washed twice in phosphate-buffered saline (PBS, Sigma-Aldrich), and pooled only briefly (to avoid proteomic alteration by allogeneic reactions) according to IRIS status (*n* = 30 IRIS, *n* = 30 non-IRIS), before being immediately flash-frozen in liquid nitrogen prior to storage at − 80 °C.

### 2.5 Protein extraction and fractionation

3 × 10^6^ frozen PBMC per group were lysed by immediate re-suspension in 3 mL phosphoprotein lysis buffer (0.25% CHAPS, 250 U benzonase, 25 mM MES pH 6.0, 1 M NaCl) at 4 °C for 30 min. Insoluble debris was pelleted by centrifugation (10,000 *g*, 30 min, 4 °C); the supernatant and cell debris were thereafter processed separately for LC-MS/MS analysis as follows.

Supernatant protein was quantitated by Bradford assay, and a phospho-purification kit (Qiagen) was employed, as per kit instructions. Briefly, supernatant was loaded onto prepared columns to capture phosphoproteins; the flow-through thus contained phospho-depleted proteins. Column-bound proteins were washed (6 mL phosphoprotein lysis buffer) and then eluted using 2 mL elution buffer (50 mM K_2_PO_4_ pH 7.5, 50 mM NaCl). Phospho-enriched and –depleted proteins were separately subjected to filter-assisted sample preparation (FASP) (Wisniewski et al., 2009). Briefly, samples were reduced, alkylated, and proteolysed (protein:trypsin ratio of 100:1) on a spin-filter membrane, facilitated by exchange of detergent-containing buffer for urea-containing buffer. Phospho-depleted peptides were further processed by high-pH reverse phase fractionation into five fractions. All peptide fractions were dried and re-suspended in 5% acetonitrile [ACN; Sigma-Aldrich], 0.1% formic acid [FA] for LC-MS/MS (Fig. 1b).

Insoluble post-lysis cell debris was re-solubilised in 100 μL 1 × sample application buffer (40 mM Tris-HCl pH 6.8, 2% SDS, 2 mM β-mercaptoethanol, 4% glycerol, 0.01% *w*/*v* bromophenol blue) at 95 °C for 10 min and loaded onto a 10% glycine-SDS gel. Proteins were separated by electrophoresis (20 mA, 90 min) and stained (colloidal Coomassie blue) prior to in-gel tryptic proteolysis, essentially as described by Shevchenko et al. (2007). Briefly, each gel lane was cut into five equal-sized slices (fractions) which were then cubed. Each fraction was washed 2 × with water (300 μL, 10 min), 1 × with 50% HPLC-grade ACN (Sigma-Aldrich; 300 μL, 10 min), incubated 3 × in 50 mM ammonium bicarbonate (300 μL, 10 min), and dehydrated in 100% ACN (300 μL) before drying *in vacuo*. Fractions were then reduced (120 μL 10 mM DTT, 1 h, 57 °C), washed (300 μL 50 mM ammonium bicarbonate, then 300 μL 50% ACN), alkylated (120 μL 55 mM iodoacetamide, 1 h in the dark, room temperature) and washed again (300 μL 50 mM ammonium bicarbonate for 10 min, then 300 μL 50% ACN for 20 min) before drying *in vacuo*. Proteins were proteolysed (100 μL 10 ng/μL trypsin, 37 °C, overnight), and tryptic peptides were extracted (2 × 100 μL 70% ACN 0.1% FA, 30 min, room temperature) and dried before re-suspension in 5% ACN 0.1% FA.

### 2.6 LC-MS/MS

Eleven peptide fractions were analysed per group. LC-MS/MS was performed using an EASY-nLC II HPLC system (Thermo Scientific) coupled to an LTQ Orbitrap Velos mass spectrometer (Thermo Scientific) equipped with a nano-electrospray ionisation source (Fig. 1d). Peptides were loaded onto a trap column (C18, 0.1 × 20 mm, 5 μm bead size), followed by separation on an analytical column (C18, 0.075 × 100 mm, 3 μm bead size) at a flow rate of 300 nL/min as follows: 5–15% solvent B (B) over 5 min, 15–40% B over 80 min, 40–60% B over 10 min, 60–80% B over 5 min, and 80% B over 10 min (B: 0.1% FA in HPLC-grade ACN).

Data acquisition, controlled by the Xcalibur software package (Thermo Scientific), was performed in ‘Top-20’, data-dependent, positive ion mode. Precursor ion settings included resolution at 60,000, automatic gain control (AGC) target of 1 × 10^6^, and scan rage 400–2000 *m*/*z*. Selected precursor ions were fragmented in the linear ion trap *via* collision-induced dissociation. Dynamic exclusion was set at 60 s to minimise repeated sequencing of the same precursors. A lock mass of 445.120025 *m*/*z* was used.

### 2.7 Bioinformatic analysis

The .RAW data files were converted to mzXML format using the Trans-Proteomic Pipeline (Deutsch et al., 2010). Crux-Barista (version 1.37 (Park et al., 2008, Spivak et al., 2009)) software mapped MS/MS fragmentation spectra to a UniProtKB Human Reference Proteome FASTA file (Consortium, 2014) to identify peptide-spectrum matches (PSMs). Search parameters included one missed cleavage and carbamidomethylation of cysteine as a fixed modification. Decoy searches performed in Crux-Barista facilitated calculation of *q*-values for identified peptides; quantitation was by spectral counting using the normalised spectral abundance factor (NSAF) method (Fig. 1e). In MS and MS/MS modes respectively, mass ranges were set to 400–3500 Da with a mass tolerance 10 ppm, and 200–3500 Da, tolerance 0.5 Da. The *q*-value threshold was set at 0.05 and protein identification required at least one high-confidence unique peptide. Where different proteins could not be distinguished based on peptide evidence (*e*.*g*. splice variants), these were gathered into a ‘protein group’ for further analysis. Computation was performed using facilities provided by the University of Cape Town ICTS High Performance Computing team (http://hpc.uct.ac.za).

NSAF values of non-redundant protein groups were used to normalise each test condition to baseline within a group. The resulting values were compared both between conditions and between groups by generating per-protein expression ratios and thus fold-changes. Within-group normalisation of NSAF values facilitates analysis of test condition influence on cell responses relative to baseline. Significant differential expression was defined as fold-changes greater than two standard deviations from the mean fold change for all protein groups. Fold-changes were log_2_ transformed for subsequent analysis using the Ingenuity Pathway Analysis (IPA) suite (Ingenuity® Systems) to identify biological canonical pathways with significantly over-represented members in the datasets.

To determine the differential proteomic response to Mtb antigens, Mtb-stimulated IRIS and non-IRIS PBMC datasets were compared, at the levels of fold-change from baseline, pathway enrichment (-log(*p*-values)), and pathway activation (z-score).

To determine the manner in which dexamethasone modulates this response, datasets for Mtb-stimulated IRIS PBMC in the absence and presence of dexamethasone were compared, at the levels of fold-change from baseline and pathway activation. To improve confidence in *in vivo* applicability of findings, *ex vivo* differences between unstimulated IRIS and non-IRIS PBMC were also independently compared (using alternate software approaches) at the level of pathway activation.

## 3 Results

Differential proteomic profiling revealed significant differences in proteomic responses to Mtb antigens in two different experimental comparisons: (1) IRIS *vs*. non-IRIS PBMC, and (2) IRIS PBMC in the presence/absence of dexamethasone. This facilitated generation of hypotheses regarding the mechanisms underlying IRIS pathogenesis and corticosteroid-based symptom alleviation.

### 3.1 Patient characteristics

 (See Table 1.)

**Table 1:** Patient clinical characteristics.

	IRIS	Non-IRIS
Males	19	15
Females	21	15
Time to ART (weeks)	6.9 ± 3.9	5.5 ± 2.5
CD4 nadir	70.68 ± 61.6	80.96 ± 74.85
VL at baseline (× 10^6^)	1 ± 1.2	1.4 ± 3.1
Time to IRIS (days)	14.4 ± 10	–
Prednisone-treated	7	7
Total	30	30

VL = viral load.

### 3.2 Protein identification, quantitation, and comparisons

A total of 701 and 911 non-redundant PBMC protein groups were identified from IRIS and non-IRIS samples, respectively (Table 2). Proteins that could be identified and quantified in test conditions and at baseline (Table 3a) were used to generate expression ratio values for input into IPA. Significantly differentially expressed proteins are presented as ranked lists with fold-changes from baseline (Table 3b and c).

**Table 2:** Proteins identified per condition and group.

Group	Condition	Number of proteins/condition	Number of proteins/group
IRIS	Baseline	467	701
	Mtb-only	692	
	Mtb + dex	695	
Non-IRIS	Baseline	698	911
	Mtb-only	635	

dex = dexamethasone.

**Table 3:** Non-redundant shared and differentially-regulated proteins.

(a) Non-redundant proteins identifiable and quantifiable between baseline and test conditions for both groups. dex = dexamethasone.			
Group	Baseline condition	Test condition	Number of proteins shared between conditions
IRIS	No stimulation	Mtb only	320
	No stimulation	Mtb + dex	337
Non-IRIS	No stimulation	Mtb only	391

(b) Ranked list: top ten proteins significantly up-regulated from baseline. FC = fold-change.						
Rank	Non-IRIS Mtb-only		IRIS Mtb-only		IRIS Mtb + dex	
	Protein (gene)	FC from baseline	Protein (gene)	FC from baseline	Protein (gene)	FC from baseline
1	P04114 (APOB)	7.080	P49327 (FASN)	11.269	P25325 (MPST)	16.228
2	P11310-2 (ACADM)	5.270	Q9BSJ8-2 (ESYT1)	8.118	O00231 (PSMD11)	12.200
3	O43143 (DHX15)	4.697	P11216 (PYGB)	6.235	Q13418 (ILK)	10.660
4	Q9UHD8-2 (SEPT9)	4.663	O00160 (MYO1F)	6.053	Q9NQC3-2 (RTN4)	6.640
5	P01040 (CSTA)	3.624	Q14766 (LTBP1)	5.798	Q9BSJ8-2 (ESYT1)	5.037
6	Q8NBS9 (TXNDC5)	3.354	Q27J81-2 (INF2)	5.562	P49407-2 (ARRB1)	4.802
7	Q9BVC6 (TMEM109)	3.326	O76074-2 (PDESA)	4.694	P60953 (CDC42)	4.276
8	P15153 (RAC2)	3.319	Q9ULV4^a^ (CORO1C)	4.301	Q07960 (ARHGAP1)	4.183
9	P61225 (RAP2B)	3.258	Q15233^a^ (NONO)	4.288	P11216 (PYGB)	4.135
10	P68871 (HBB)	2.887	Q9Y4D1-2^a^ (DAAM1)	4.259	Q27J81-2 (INF2)	4.069

(c) Ranked list: top ten proteins significantly down-regulated from baseline. FC = fold-change.						
Rank	Non-IRIS Mtb-only		IRIS Mtb-only		IRIS Mtb + dex	
	Protein (gene)	FC from baseline	Protein (gene)	FC from baseline	Protein (gene)	FC from baseline
1	P30084 (ECHS1)	− 4.664	Q16666-2 (IFI16)	− 7.450	P12259 (F5)	− 9.885
2	P31946-2 (YWHAB)	− 3.760	P14314 (PRKCSH)	− 6.531	P48426 (PIP4K2A)	− 6.329
3	P50991 (CCT4)	− 3.672	P13667 (PDIA4)	− 5.685	P13667 (PDIA4)	− 6.061
4	Q06323 (PSME1)	− 3.619	Q9H082 (RAB33B)	− 5.651	Q6UX06 (OLFM4)	− 5.265
5	P30041 (PRDX6)	− 3.570	Q8NBS9 (TXNDC5)	− 5.049	P13804 (ETFA)	− 5.114
6	Q92888-3 (ARHGEF1)	− 3.426	P08133 (ANXA6)	− 4.981	D6RF44 (HNRNPD)	− 5.082
7	Q02218 (OGDH)	− 3.340	P07359 (GP1BA)	− 4.856	Q9Y230 (RUVBL2)	− 4.957
8	E7EVX7 (ANK1)	− 2.855	P06753 (TPM3)	− 4.733	O14818 (PSMA7)	− 4.839
9	P22695^a^ (UQCRC2)	− 2.777	P02656 (APOC3)	− 4.662	P34932 (HSPA4)	− 4.155
10	P04908^a^ (BCAP31)	− 2.698	F5H2R5^a^ (ARHGDIB)	− 4.312	Q5T4S7 (UBR4)	− 3.907

a Relatively low statistical significance, but high fold-change.

a Relatively low statistical significance, but high fold-change.

**Fig. 2:**
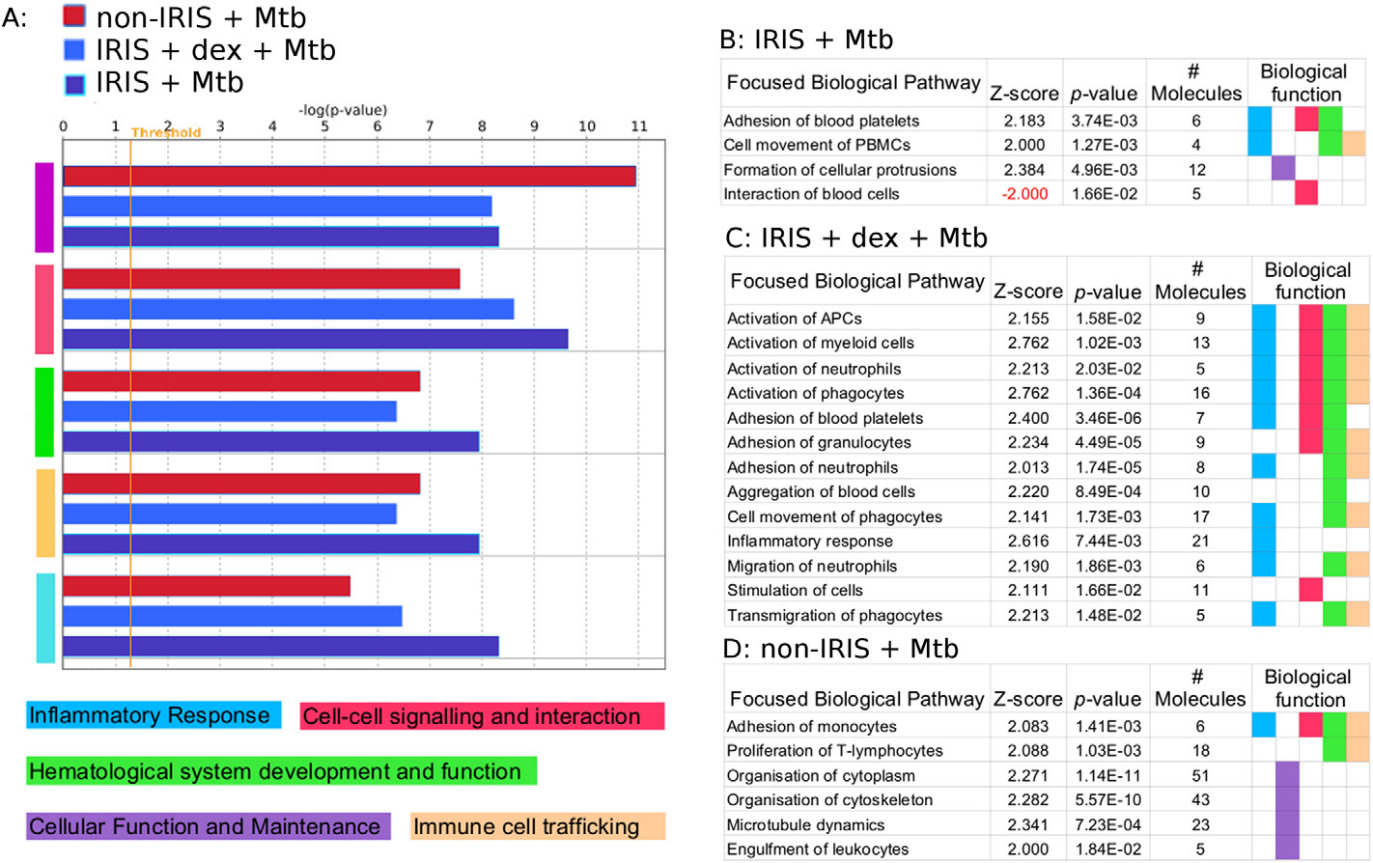
Dexamethasone modulates IRIS-group PBMC responses to Mtb antigens to resemble those of non-IRIS-group PBMC. (a) Changes in enrichment significance (orange line: *p* = 0.05) for all three datasets (dark blue indicates non-IRIS Mtb-only, aqua indicates IRIS Mtb-only, and green indicates IRIS Mtb + dexamethasone) across five broad biological functions. (b)–(d) Focused analysis of significantly-activated pathways.

Key functional pathways were significantly differentially enriched across datasets (colour-coded in Fig. 2a). Activation scores of more focused biological functions (Fig. 2b-d) indicate modulation of the type of response that PBMC are capable of.

### 3.3 Characteristics of non-IRIS PBMC proteomic responses to Mtb antigens

At the level of fold-change from baseline, members of the top 10 differentially regulated proteins in the non-IRIS group play prominent roles in key canonical pathways, such as Rac, RhoGDI, and integrin signalling, as well as F_c_γ receptor (F_c_γR)-mediated phagocytosis.

**Fig. 3:**
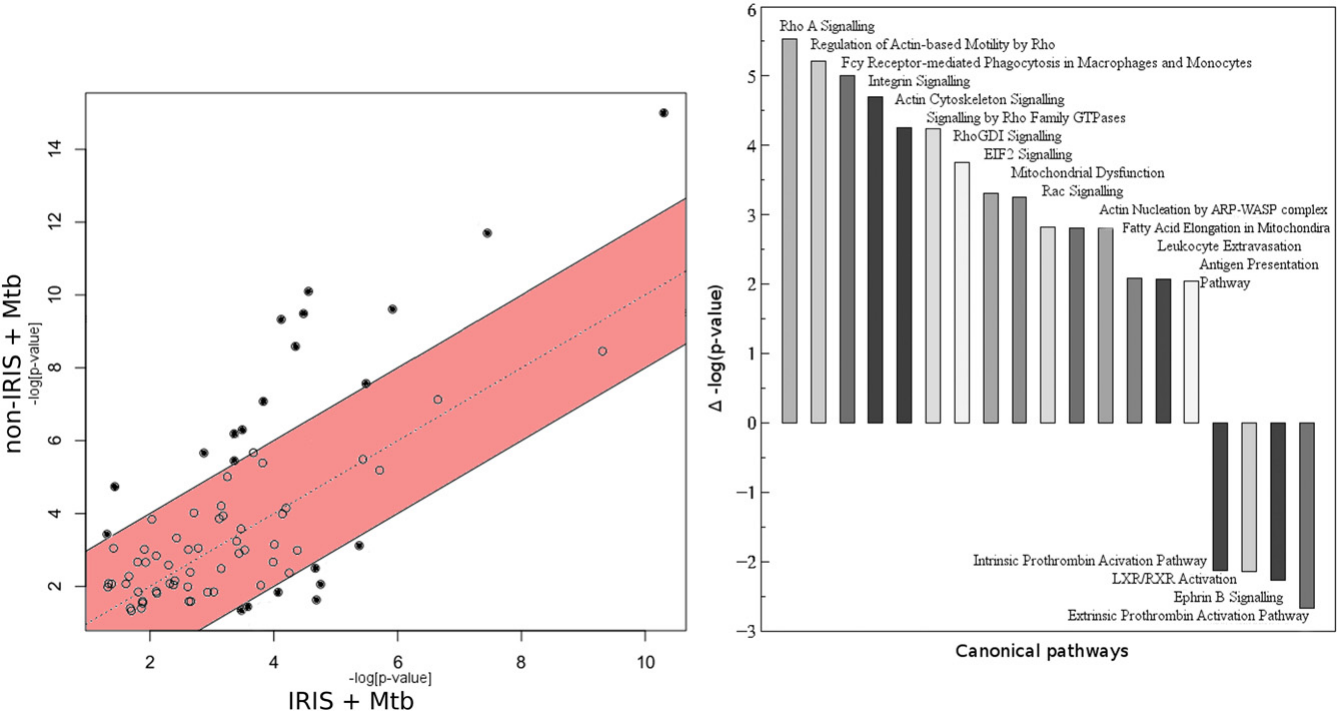
Significantly enriched canonical pathways differ between groups. Left: –log(*p*-values) from IRIS and non-IRIS datasets plotted against each other (pathways outside the standard-deviation band (*p* = 0.01) are considered significantly differentially represented). Right: significantly differentially represented pathways plotted on a differential bar chart (upper bars positively associated with non-IRIS group; lower bars positively associated with IRIS group).

At the level of pathway enrichment, non-IRIS PBMC responses encompass more pathways that are significantly enriched: when –log(*p*-values) are plotted to identify significantly differentially enriched pathways between the IRIS and non-IRIS datasets, consistently higher levels of significant enrichment are seen in the non-IRIS group (Fig. 3, left).

At the level of pathway activation, fewer pathways are active during non-IRIS PBMC responses to Mtb antigens, but many achieve significant activation scores (Fig. 2d). Many of the significantly-activated pathways are associated with both innate and adaptive immune functions.

**Fig. 4:**
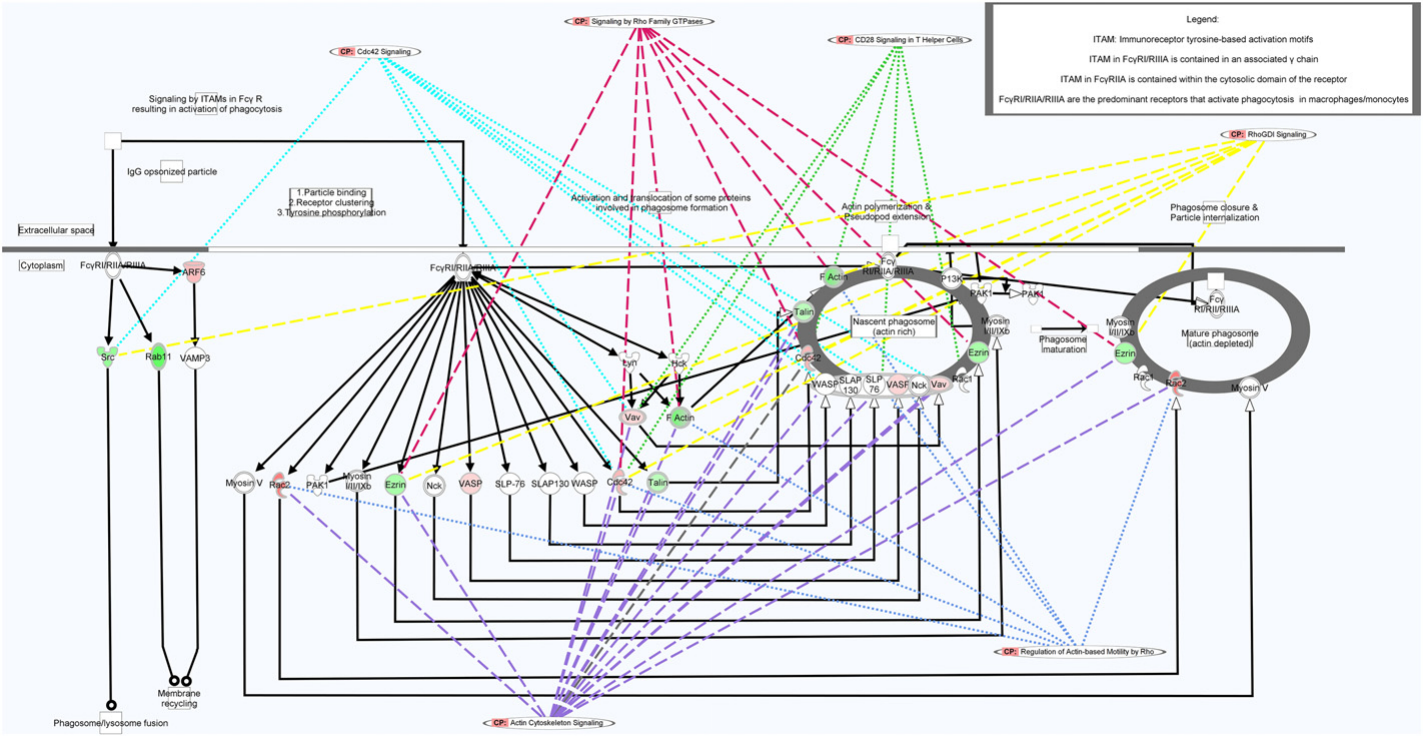
FcγR-mediated phagocytosis canonical pathway overlaid with related pathways to illustrate integration of various pathways *via* key regulatory proteins (inset: number of pathways regulated by each protein).

Non-IRIS group proteins contributing to enrichment, activation, and efficacy of the F_c_γR-mediated phagocytic pathway are also relevant to other actin cytoskeletal dynamics-related pathways, and some even act as regulators in several different pathways (Fig. 4): enriched pathways are tightly functionally integrated (linked *via* overlapping regulatory mechanisms or roles in actin cytoskeletal dynamics).

### 3.4 Characteristics of IRIS PBMC proteomic responses to Mtb antigens

At the level of fold-change from baseline, TXNDC5 is down-regulated in IRIS relative to non-IRIS PBMC. At the level of pathway enrichment, only four pathways are identified as significantly enriched in the IRIS group (Fig. 3, right), and the canonical pathway for antigen presentation is significantly enriched in the non-IRIS group. Although the eIF2 canonical pathway is relatively more enriched in the non-IRIS group, at the level of pathway activation, it is relatively more up-regulated in the IRIS group. Activation of one critical pathway (Interaction of Blood Cells) is even relatively inhibited in the IRIS group (Fig. 2b). Many more immune and inflammation-related pathways are activated in the IRIS group than the non-IRIS group. Additionally, higher activation scores are observed for the FcγR-mediated phagocytosis pathway in non-IRIS (relative to IRIS) PBMC.

### 3.5 Modulation of these responses by dexamethasone

At the level of fold-change from baseline during IRIS PBMC responses to Mtb antigens, dexamethasone differentially regulates proteins associated with pathways more closely resembling those identified as enriched during non-IRIS PBMC responses to Mtb antigens. At the level of pathway activation, dexamethasone also increases the number of activated pathways in the IRIS group that achieve significance, and many of these pathways are associated with innate immune responses (Fig. 2c).

**Fig. 5:**
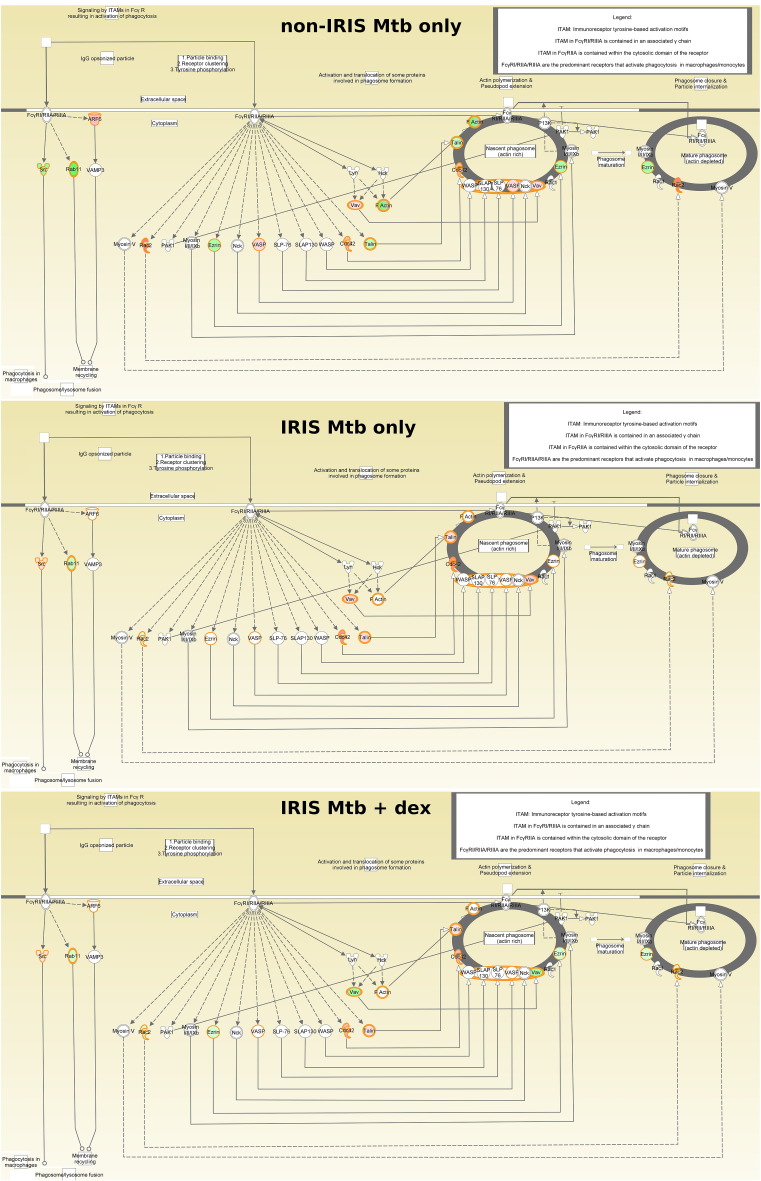
F_c_γR-mediated phagocytosis canonical pathway utilisation per condition. Normalised NSAF values are overlaid on the F_c_γR-mediated phagocytosis canonical pathway, with colours representing fold-change from baseline (red = up-regulated, green = down-regulated).

Even when dexamethasone is present during Mtb stimulation of IRIS PBMC, Mtb-stimulated non-IRIS PBMC still display relatively higher activation scores for the FcγR-mediated phagocytosis pathway (Fig. 5). The pattern of relatively increased FcγR-mediated phagocytosis pathway activation in the non-IRIS (relative to the IRIS) group persists when comparing *ex vivo* proteomic differences at baseline (z = 3.6).

## 4 Discussion

Here, a label-free proteomic discovery strategy identified and quantified 701 IRIS and 911 non-IRIS PBMC unique protein groups. In principle, a discovery proteomic study of paradoxical TB-IRIS has the capacity to reveal pathogenic mechanisms not accessible to immunological studies that assess a limited number of known secreted or surface proteins. Biological pathway analyses based on our unbiased data revealed clear differences in the response to Mtb antigens between IRIS and non-IRIS groups, as well as significant modulation of IRIS PBMC responses by dexamethasone. These differences may be at least partly responsible for the IRIS phenotype and corticosteroid-mediated symptom alleviation, leading to hypotheses regarding biological mechanisms underlying these phenomena.

Patient-derived PBMC were used as a patient-specific model of IRIS pathogenesis, representing the immune cell types likely involved in this process, while dexamethasone was used as a stable analogue of prednisolone - itself the pro-drug of prednisone.

An important caveat to our approach is that, since blood used in this study was taken three weeks post-ART initiation, conclusions can only be drawn regarding IRIS pathogenesis, not predisposition. A further caveat is that limiting clinical sample quantities necessitated use of a sample pooling strategy. Pooling is controversial because it precludes identification of outliers contributed by any single sample; however, levels of an individual protein in a pool by definition represent the average expression of those proteins in the samples making up the pool, and proteins detectable in individual samples are usually also detectable in the pool (Diz et al., 2009). Pooling additionally reduces the major contributor to variance (biological variance), thereby increasing power: the averaging effect of pooling makes the most evident differences and similarities between groups easier to detect (Diz et al., 2009, Oberg and Vitek, 2009). Exhaustive cell counts at every step of processing aimed to minimise technical bias in contribution from any one sample to the pool.

An additional caveat in interpretation of our results is that - for technical reasons - for the IRIS sample insoluble protein fractions, the pool consisted of six samples rather than 30, which may have led to relatively fewer proteins being identified in the IRIS group. However, despite this, the total number of non-redundant proteins identified here represents ~ 25% of the currently observable PBMC proteome (Končarević et al., 2014) and likely represents the most abundant cellular proteins (most of the dry protein mass). This extent of proteome coverage proved sufficient to observe multiple proteins from key differentially-regulated biological pathways, enabling canonical pathway enrichment analysis as a means to generate biologically sound mechanistic hypotheses for validation.

Our findings regarding non-IRIS PBMC proteomic responses to Mtb antigens suggest improved consensus and co-ordination of response pathways, executed by well-organised cells. The majority of enriched pathways are tightly functionally integrated, being linked *via* overlapping regulatory mechanisms or roles in actin cytoskeletal dynamics. Additionally, most are pre-requisites for effective protective immunity: since actin cytoskeletal dynamics are a key determinant of PBMC function (including signal transduction, motility, phagocytosis and antigen clearance, antigen presentation, cellular interaction, cellular homeostasis, and apoptosis, among others), appropriate functioning of this system is by extension crucial to combating infection and resolving inflammation. Additionally, many significantly-activated pathways are associated with both innate *and* adaptive immune functions.

*Motility* is primarily regulated and integrated by the Rho family of small GTPases (*e*.*g*. RhoA, Rac, Cdc42), involved in key canonical pathways significantly over-represented in the non-IRIS group. This suggests non-IRIS PBMC have a preserved capacity to co-ordinate and regulate motility for access to and egress from sites of infection, as well as phagocytosis. Indeed, TNFα – up-regulated in IRIS patient blood – is known to induce monocyte/macrophage hypercytokinesis (increased but undirected motility) (Jones, 2000), which may impair antigen clearance.

Actin reorganisation is also required for *phagocytosis*: reorganisation is triggered by receptor ligation and G protein (*e*.*g*. Rac, Cdc42) signalling, including activation of the ARP 2/3 complex (Fig. 4), while internalization is controlled by a molecular complex containing WASP. It is notable that the canonical pathway for *antigen presentation* - required for adaptive response activation - is significantly enriched in the non-IRIS group, while no adaptive-associated pathway activation is observed in the IRIS group.

Taken together, our data suggest that non-IRIS PBMC display preservation of functionalities required for protective immunity, including those related to appropriate cytoskeletal reorganisation, and both arms of the immune response.

By contrast, our findings regarding IRIS PBMC proteomic responses to Mtb antigens suggest the novel idea of poor consensus and co-ordination of response pathways (*i*.*e*. few activated pathways achieve significance), possibly due to relatively deficient intercellular interaction (relative inhibition of the Interaction of Blood Cells pathway), or pre-existing cellular fatigue (due to *e*.*g*. over-activation or a relative deficiency of trophic factors experienced *in vivo*).

In support of the notion of fatigue, TXNDC5 (down-regulated in the IRIS group), an endoplasmic reticulum lumen protein, is associated with suppression of apoptosis in endothelia (Bruneel et al., 2005) and other human cells. Additionally, the eIF2 canonical pathway - involved in protein synthesis and cellular response to stress – is relatively more activated in the IRIS group, favouring apoptosis. These findings suggest that IRIS PBMC (*e*.*g*. monocytes) may be more prone to *in situ* apoptosis, instead of efferocytosis and antigen clearance.

While pathways enriched and activated in the IRIS group are apparently not tightly integrated, they are at least functionally linked, as expected in a setting of inflammation. Concordant with literature, the overall inflammatory response magnitude appears higher in the IRIS dataset, with many more immune and inflammation-related pathways activated.

Interestingly, the apparent lack of significant involvement of adaptive response-associated pathways in the IRIS group may suggest deficient adaptive sustenance of the innate activation required for successful destruction of Mtb by phagocytes. While HIV infection leads to T-cell depletion and functional exhaustion, *in vivo* development of TB-IRIS is associated with antigen-specific T-cell expansion (Bourgarit et al., 2009, Meintjes et al., 2008b), which is seemingly at odds with our proteomic data. We cannot yet explain this apparent contradiction, but note that inappropriate T-cell *activity* or reduced innate cell responsiveness to adaptive signals remain possibilities. Taken together, however, our data suggest that IRIS-group PBMC responses, though vigorous, may be too uncoordinated to effectively clear Mtb antigens, leading to inappropriate inflammation.

Our data also show that dexamethasone markedly alters IRIS-group PBMC responses to Mtb antigens *in vitro*: instead of multiple IRIS-group activated pathways with few achieving statistical significance, in the presence of dexamethasone the number of significantly activated pathways associated with innate immune responses is increased. This suggests that dexamethasone partially ameliorates IRIS-group PBMC dysfunction by restoring a more unified response (although centred on innate rather than both innate and adaptive response-associated pathways). This is consistent with the idea of deficient innate activation by adaptive signals and is also consistent with the observation that ~ 20% of corticosteroid-treated TB-IRIS patients relapse after prednisone withdrawal (Meintjes and Sonderup, 2011). Molecular mechanisms by which dexamethasone achieves these effects are not immediately apparent from our data, but steroids generally suppress pro-inflammatory T-cell activity, while inducing regulatory T-cell and tolerogenic dendritic cell activity (Zen et al., 2011). At low, physiological-range levels, steroids can also enhance macrophage phagocytic and efferocytotic capability (McColl et al., 2007), providing a further potential route for dexamethasone to modulate the IRIS-group PBMC response to Mtb antigens. Taken together, our data therefore suggest that dexamethasone partially rescues the uncoordinated IRIS-group phenotype, either by dampening adaptive pathways to unmask focused innate pathways, or by directly boosting innate pathways, perhaps promoting local antigen clearance.

Notably, our proteomic data point to key differences in utilisation of the F_c_γR-mediated phagocytosis pathway between IRIS- and non-IRIS-group PBMC. F_c_γR are expressed on phagocytes, and ligation by IgG-opsonised particles or immune complexes (IC) leads to signalling *via* immunoreceptor-based tyrosine activation motifs (ITAMs). Whole-blood gene expression studies have identified F_c_γRIA (a high-affinity IgG receptor mainly expressed on monocytes/macrophages) and F_c_γRI signalling as classifiers of active TB, indicating their likely importance in normal TB pathogenesis (Sutherland et al., 2014, Maertzdorf et al., 2011, Berry et al., 2010). This agrees with findings of significantly reduced F_c_γRIA expression following successful TB treatment (Sutherland et al., 2014, Cliff et al., 2013). Non-opsonic or surfactant-mediated phagocytosis is thought to be important early in Mtb infection (Schafer et al., 2009), and complement-mediated opsonisation is critical (when C3 is absent, phagocytosis of Mtb by monocytes/macrophages declines by 70–80% (van Crevel et al., 2002)). However, complement-mediated uptake induces a relatively non-robust Th response, while F_c_γR-mediated uptake of Mtb strongly “activates the antimicrobial, degradative and … antigen-presentation activities of macrophages and dendritic cells to enhance the Th1 response” (Igietseme et al., 2004). Maertzdorf et al. (2011) also found exacerbated expression of complement in tuberculosis, which is consistent with the findings of Tran et al. (2013) that inappropriate control of complement activation may contribute to TB-IRIS.

The higher F_c_γR-mediated phagocytic pathway activation observed here in the non-IRIS group - including in our ‘baseline-only’ comparison of IRIS- and non-IRIS-group PBMC - therefore suggests a novel, testable hypothesis that a relative deficit in F_c_γR-mediated phagocytosis occurs in TB-IRIS patient PBMC *in vivo*, and that this directly affects innate cell-mediated antigen clearance capacity, thereby contributing to higher Mtb antigen loads and aberrant immune responses during TB-IRIS. Whether the deficient F_c_γR-mediated phagocytosis response observed here in IRIS-group PBMC is a manifestation of simple fatigue, B-cell dysfunction, or an as yet unrecognised factor remains to be determined.

In summary, our data suggest that TB-IRIS patients may take up, present, and clear Mtb antigens less effectively, resulting in an increased antigen load that contributes to the TB-IRIS phenotype through inappropriate or prolonged stimulation of inflammatory cascades after ART initiation. In particular, our data suggest that IRIS-group PBMC respond to Mtb antigens in a dysregulated manner *in vitro*, with possible underlying mechanisms of dysfunction including: cellular fatigue (including *in situ* apoptosis), unfocused/uncoordinated cellular responses (possibly related to poor cellular interaction), dysregulated motility, deficient FcγR-mediated phagocytosis, and lack of activating adaptive signals (or lack of innate responses to these signals). Dexamethasone ameliorates this IRIS-group PBMC dysregulation *in vitro* by modulating functions to resemble those of non-IRIS-group PBMC (*e*.*g*. by unmasking or boosting innate response pathways), suggesting that alternative therapeutic strategies for TB-IRIS may yet prove more effective than the currently used prednisone. By contrast, our data suggest that non-IRIS-group PBMC display proteomic characteristics consistent with known immune responses to TB infection, including preservation of functionality required for protective immunity, including those related to appropriate cytoskeletal reorganisation and both innate and adaptive immune responses. Overall, our proteomic findings are thus generally consistent with the literature, including the model of Barber et al. (2012), but provide new insight into plausible underlying molecular mechanisms. Experiments to validate these hypotheses are currently underway.

## Funding sources

JMP was supported by the South African Medical Research Council (SA MRC) through its National Health Scholars Programme. JMB was supported by a South African Research Chair grant from the National Research Foundation (NRF) of South Africa (grant no. 64760). GM was supported by the Wellcome Trust (098316), a South African Research Chair grant from the NRF (grant no. 64787), NRF incentive funding (UID 85858), and the SA MRC through its TB and HIV Collaborating Centres Programme with funds received from the National Department of Health (RFA# SAMRC-RFA-CC: TB/HIV/AIDS-01-2014). RJW was supported by the Wellcome Trust (084323, 088316), the SA MRC (U1175.01.001.00014.02), the European Union (FP7-Health-F3-2012-305578, FP7-People-2011-IRSES), and the NRF (96841).

## Conflict of interest statement

The authors declare that there are no conflicts of interest. The funders had no role in the study design, data collection, data analysis, data interpretation, or writing of this report. The opinions, findings, and conclusions expressed in this manuscript reflect those of the authors alone.
